# Characterization and Comparative Profiling of MiRNA Transcriptomes in Bighead Carp and Silver Carp

**DOI:** 10.1371/journal.pone.0023549

**Published:** 2011-08-15

**Authors:** Wei Chi, Chaobo Tong, Xiaoni Gan, Shunping He

**Affiliations:** 1 Laboratory of Fish Phylogenetics and Biogeography, Institute of Hydrobiology, Chinese Academy of Sciences, Wuhan, Hubei, People's Republic of China; 2 Graduate University of the Chinese Academy of Sciences, Beijing, People's Republic of China; University of Georgia, United States of America

## Abstract

MicroRNAs (miRNAs) are small non-coding RNA molecules that are processed from large ‘hairpin’ precursors and function as post-transcriptional regulators of target genes. Although many individual miRNAs have recently been extensively studied, there has been very little research on miRNA transcriptomes in teleost fishes. By using high throughput sequencing technology, we have identified 167 and 166 conserved miRNAs (belonging to 108 families) in bighead carp (*Hypophthalmichthys nobilis*) and silver carp (*Hypophthalmichthys molitrix*), respectively. We compared the expression patterns of conserved miRNAs by means of hierarchical clustering analysis and log2 ratio. Results indicated that there is not a strong correlation between sequence conservation and expression conservation, most of these miRNAs have similar expression patterns. However, high expression differences were also identified for several individual miRNAs. Several miRNA* sequences were also found in our dataset and some of them may have regulatory functions. Two computational strategies were used to identify novel miRNAs from un-annotated data in the two carps. A first strategy based on zebrafish genome, identified 8 and 22 novel miRNAs in bighead carp and silver carp, respectively. We postulate that these miRNAs should also exist in the zebrafish, but the methodologies used have not allowed for their detection. In the second strategy we obtained several carp-specific miRNAs, 31 in bighead carp and 32 in silver carp, which showed low expression. Gain and loss of family members were observed in several miRNA families, which suggests that duplication of animal miRNA genes may occur through evolutionary processes which are similar to the protein-coding genes.

## Introduction

MicroRNAs (miRNAs) are a family of small, non-coding RNAs of approximately 22 nucleotides (nt) in length, derived from 60 to 80-nt-long stem-loop precursors that are abundant in nearly all metazoans, plants and even viruses [1]–[4]. By modulating the stability and translational efficiency of target mRNAs, miRNA plays a key role in regulating the expression of genes, which influences a range of physiological processes, including metabolism, apoptosis, development of the nervous system, immunity defense, and pathogenesis of cancer [5]–[7]. Since the miRNA (*lin-4)* was discovered in *Caenorhabditis elegans* in 1993 [8], extensive research has been undertaken focusing on the biosynthesis, functions and the mechanisms of action of miRNAs. During the biogenesis of animal miRNA, one RNA duplex is released from the precursor transcript after a two-step splicing by the RNase III enzymes Drosha and Dicer. One strand of the duplex, known as mature miRNA, is incorporated into the RNA-induced silencing complex (RISC) to exert its functions in association with Argonaute proteins [9], while the complementary strand, known as a star sequence, is degraded. However, Okamura et al. showed that the star sequence may also be functional [10]. After binding to a target mRNA, the Ago-miRNA complex induces cleavage and degradation. If, however, the binding of the Ago-miRNA complex and the 3′ UTR results in the target mRNA being imperfect, this leads to translational inhibition or deadenylation and subsequent decapping and degradation of the target mRNA [9]. While the role of miRNAs was recognized early on, studies on the level of whole miRNA transcriptomes have only recently been undertaken.

In the past few years, direct cloning, sequencing and northern blot analyses have been widely used to detect and identify many individual miRNAs [11], [12]. There are, however, some limitations to these methods: the capability of detecting miRNAs in low abundance is poor, due to variable expression levels, and the specificities of precise temporal and spatial expression during developmental stages are also poor. This explains why small-scale sequencing mainly reveals conserved miRNAs, as non-conserved miRNAs are often expressed at lower level than conserved miRNAs [13], [14]. High throughput sequencing technology has made it possible to precisely identify non-conserved or weakly-expressed miRNAs, and many species-specific miRNAs have been characterized in plants, such as Arabidopsis and wheat [15], [16], and also in animals, such as fish, chicken and human [17]–[20].

Bighead carp (*Hypophthalmichthys nobilis*) and silver carp (*Hypophthalmichthys molitrix*) are two closely related species of the subfamily *Hypophthalmichthys* within Cyprinidae. Both species are endemic to East-Asia and are the most intensively–cultured species among the filter-feeding fishes, being able to filter phytoplankton and other particles as small as 4–10 µm [21], [22]. For this reason, they have been introduced into other countries, originally for the purpose of controlling algal blooms in eutrophic waters. Bighead carp and silver carp (in this paper also referred to as ‘the two carps’) are also the main commercial fishes captured and cultured in China and several other countries. There are, however, some physiological and morphological differences between these two species, such as the huge difference of the size of their skull bones. There has been abundant research into the temperature and salinity tolerance, sexual maturity and mating behavior, spawning, early development and feeding habits of the two carps [23]. Biological processes and physiological differences between these two species are related to changes at the molecular level and probably involve both transcriptional and post-transcriptional regulation of gene expression which are still poorly understood.

**Figure 1:**
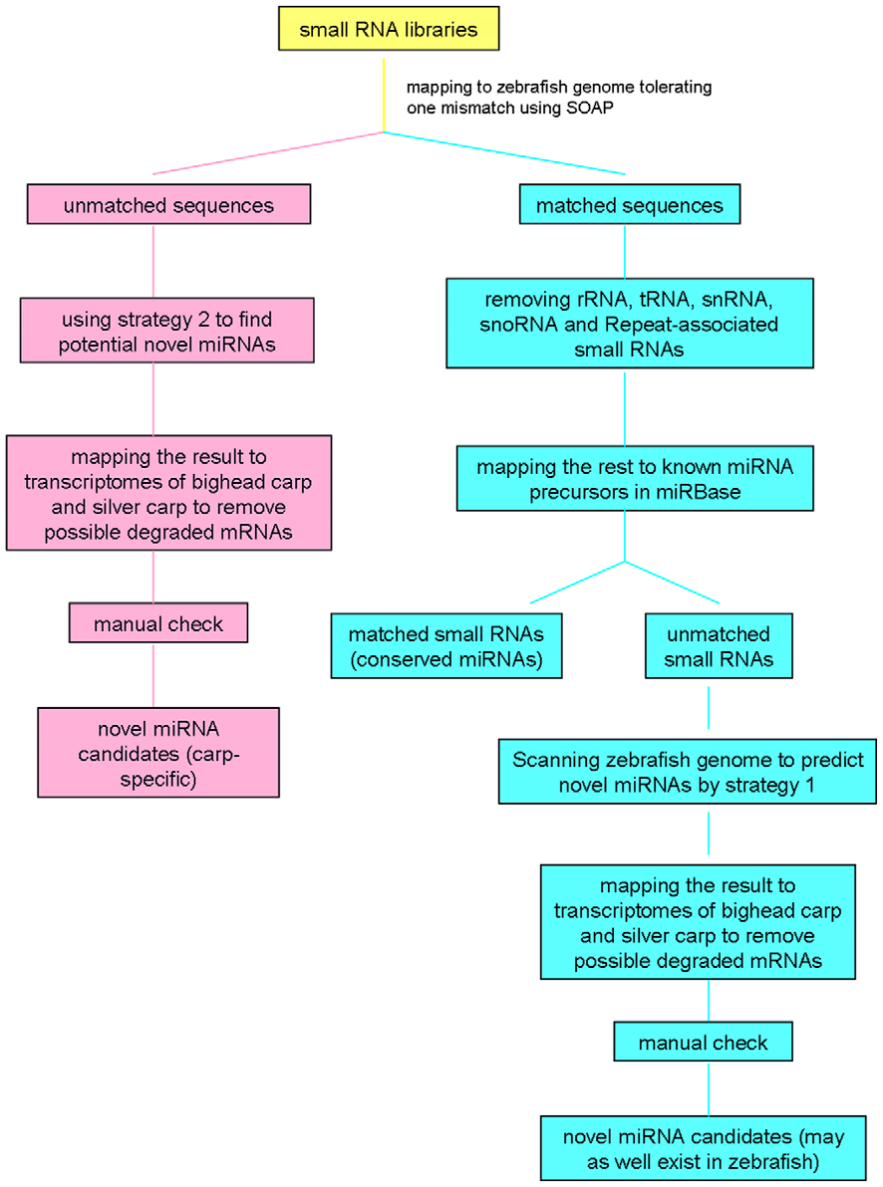
Step-by-step schematic description of the strategy for bighead carp and silver carp miRNA discovery.

In this study, we adopted the high-throughput sequencing method to characterize small RNA transcriptomes of bighead and silver carp, and an integrative strategy was followed to detect and analyze their whole microRNA transcriptomes (Figure 1). With this strategy, we identified 167 conserved miRNAs in bighead carp and 166 in silver carp, and discovered 39 novel miRNAs in bighead carp and 54 in silver carp.

## Results

### Construction of small RNA libraries

**Figure 2:**
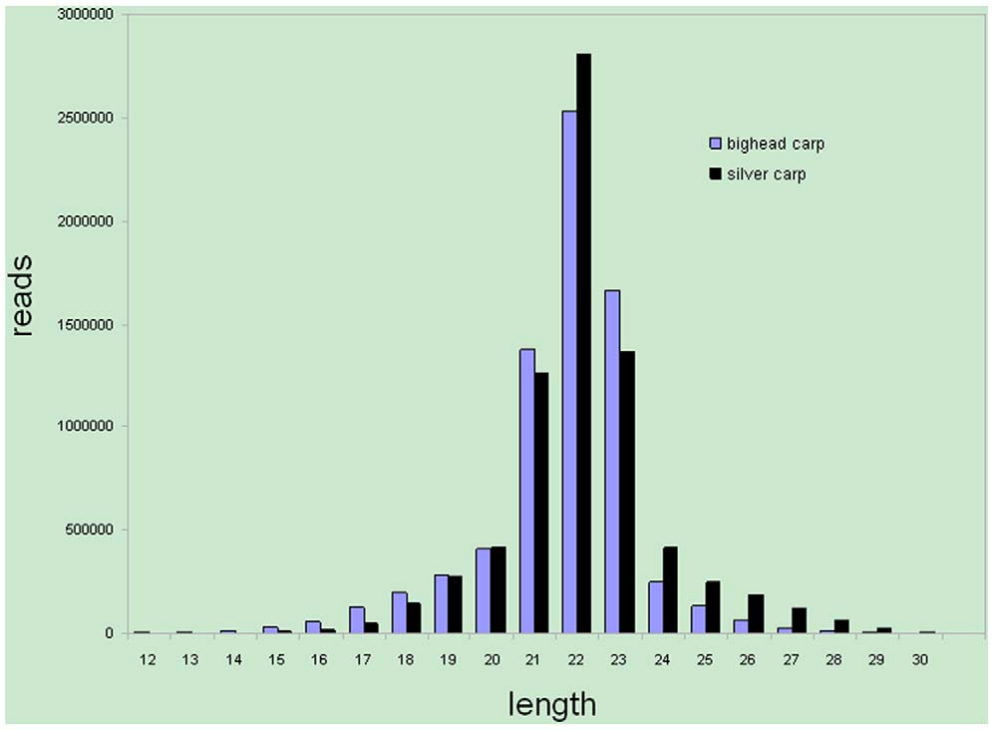
Length distribution of small RNAs in bighead carp and silver carp.

We originally obtained 8070608 reads from bighead carp (*Hypophthalmichthys nobilis*) and 8311956 reads from silver carp (*Hypophthalmichthys molitrix*). Very little difference was found in the length distribution of the sequences from the two species, most of the sequences had between 21–23 nucleotides (Figure 2). After discarding low-quality reads, 3′adaptor reads, 5′adaptor contaminants, and sequences shorter than 18 nucleotides, reads of 6966950 and 7348464 for bighead carp and silver carp, consisting of 349474 and 507077 unique sequences respectively, remained for analysis. Raw data are available at Gene Expression Omnibus (GEO: GSE22232). Given that no genome or EST databases for either bighead carp or silver carp are available, we have utilized the zebrafish genome as a reference for the analysis that followed. Data was downloaded from the UCSC database (http://hgdownload.cse.ucsc.edu/downloads.html#zebrafish). The high quality sequences were mapped to the reference genome using SOAP (http://soap.genomics.org.cn). For the selection of the computing algorithm, we chose a tolerance of one mismatch for mapping [24], which resulted in 4574654 sequences in bighead carp and 4711400 sequences in silver carp were perfectly mapped to the reference genome; 9610 and 13733 reads in bighead and silver carp, respectively, were mapped to the genome with one mismatch.

Subsequently, we performed a database search and adopted a computational strategy to assign each small RNA sequence in the mapping result with a unique annotation. The small RNAs were then classified into different categories according to their annotations. We separated out and discarded rRNA, tRNA, snRNA and snoRNA using blast against known noncoding RNAs deposited at Rfam database and NCBI genbank database (data not shown). We also discarded a tiny group of small RNAs of about 24–27 nt in length (designated as repeat-associated small interfering RNAs: rasiRNAs), which mediate the silencing of genomic repeats and transposon control [25], [26]. The remaining sequences were clustered according to sequence similarity, taking in consideration that a single miRNA gene always has different variants at sequence level due to imprecise processing by Drosha and Dicer and various biochemical modifications. We assumed that the sequence with the dominant number of reads in a cluster was likely to be the authentic sequence, due to its relatively high expression level.

### Abundant conserved microRNAs in the two carps

**Figure 3:**
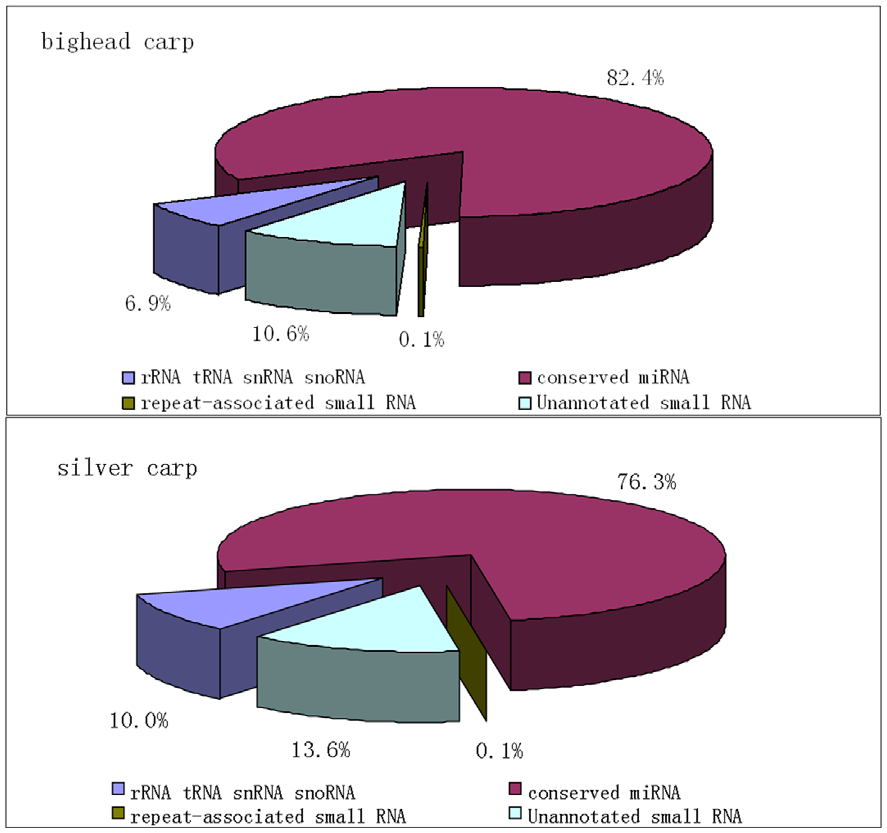
Composition of small RNA libraries in bighead carp and silver carp.

In order to identify the conserved miRNAs of these two carps, we compared the mapped sequences against currently-released mature miRNAs in miRBase [27]. In bighead carp, we have characterized 167 conserved miRNAs, belonging to 108 families and 3775832 sequences in total. In silver carp, 166 miRNAs, belonging to 108 families, were characterized, with a total of 3605148 sequences (Table S1). One hundred and sixty-four conserved miRNAs were found both in bighead carp and silver carp. Among this part of the dataset that mapped to the zebrafish genome, over three quarters belonged to miRNAs (82.3% in bighead carp, 76.3% in silver carp). In addition to these sequences, some unannotated sections were also included (Figure 3). For the miRNAs that have already been identified and validated, miR-122 has the highest expression in both species: 1808356 sequences in bighead carp and 556153 sequences in silver carp. While for some miRNAs (such as miR-725, miR-733 and miR-736), their numbers are less than 5 reads in both carps.

### Novel miRNAs prediction with different strategies

For the sequences that were not matched to known miRNA precursors, we used our first strategy (referred to as ‘strategy 1’) to detect potential novel miRNAs. This strategy is based on the observation that miRNA precursors have characteristic fold-back structures comprising two dependent parts. In the first part, by mapping the miRNA sequence of the two fishes onto the zebrafish genome, candidate miRNA sites are screened from breakpoints in the zebrafish genome. In the second part, a minimal stringent criterion is used to select miRNA candidates, which ensures that a majority of recovered miRNAs satisfy the common features of a miRNA gene (see *Materials and methods*). This approach was executed by MIREAP (https://sourceforge.net/projects/mireap/), and produced 18 candidate miRNAs in bighead carp and 29 in silver carp. Each miRNA candidate was then manually checked. The data was first mapped to the transcriptomes of the two carps which we have also sequenced (data unpublished) to remove putative mRNA fragments. We obtained a few scaffolds that matched with the candidate miRNAs, and these scaffolds were then blasted against GenBank to confirm their identities. All of them except one were identified as mRNA degradation fragments and were discarded. The remaining candidates were compared with known miRNAs in miRBase, and some matched with the known miRNAs with either two mismatches at 3′ ends or one mismatch in the middle and one or two nucleotides fluctuation in the entire length. This allowed us to gain 8 novel miRNAs in bighead carp and 22 in silver carp (Table S2). Since the prediction was based on the zebrafish genome, these miRNAs should theoretically exist in zebrafish as well, but could have escaped detection with the methodologies used.

Considering that the genome references of the two carps are not yet available, the approaches used relying on phylogenetic conservation of structure and sequence cannot adequately identify novel miRNAs. To perform deep mining of the dataset, we used a computational method (referred to as ‘strategy 2’) to search for miRNA duplex-like pairs from the part of our dataset that was not mapped to the genome of zebrafish. This computational method is based on high-throughput sequencing but does not require the availability of whole genome sequencing data. The detailed perl script and criteria are described in *Materials and methods*. The results were mapped to the transcriptomes of the two carps (data unpublished) to eliminate mRNA degradation products and a manual check was performed in the remaining sequences. We finally obtained 31 putative novel miRNAs in bighead carp and 32 in silver carp. For these candidate miRNAs, we could not identify homologs in zebrafish or any other species. Eleven of them were found in both carps, while 20 were found in bighead carp and 21 existed in silver carp only (Table S3).

### Conservation of miRNA*

Studies have demonstrated that miRNAs play an important role in animal development, and many of them are highly conserved, even between vertebrates and invertebrates. However, miRNA* may not be so conserved because although miRNA and miRNA* are complementary, their base-pairing is not perfect (for instance, bulges exist and GU pair is allowed). The miRNA* are not as stable as miRNA and usually are poorly detected in high throughput data sets or may even be absent. In the profiling of our dataset, we have obtained several miRNA*s in the data sets of the two carps. Analysis confirmed that they were homologs of zebrafish and human miRNA*, and the majority of them were as conserved as mature miRNAs (Table S4). Most of the miRNA*s were detected at low level, except for hno-miR-1388* and hmo-miR-1388*. Interestingly, in the library of conserved miRNAs, only 671 and 488 miR-1388 sequences were found in bighead and silver carp, respectively, while higher number of miR-1388* than miR-1388 reads were detected. Conversely, highly expressed miRNAs like miR-122 and miR-192 were poorly identified by their star sequences. Generally, miRNA star sequences were assumed to be carrier strands without any particular function. Recent studies have, however, shown that miRNA*s in *Drosophila melanogaster*, although few in number, can associate and function with Argonaute proteins [10]. Therefore, the relatively high number of reads of miR-1388* suggest that it may play a functional role in regulating gene expression.

**Figure 4:**
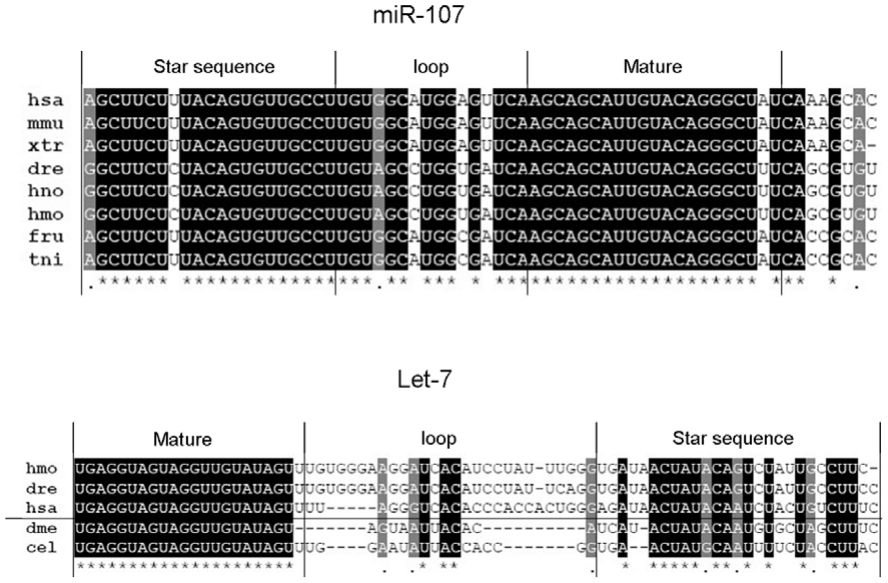
Two examples of conservation of miRNA precursors. miR-107 was compared among seven vertebrates; let-7 was compared across vertebrates and invertebrates. Alignments were performed by Mega 4.0. has: *H. sapien*; mmu: *M. musculus*; xtr: *X. tropicalis*; dre: *D. rerio*; hno: *H. nobilis*; hmo: *H. molitrix*; fru: *F. rubripes*; tni: *T. nigroviridis*; dme: *D. melanogaster*; cel: *C. elegans*.

To further study the miRNA* identified, we have stochastically cloned and sequenced a few precursors of miRNA in bighead and silver carp. Figure 4 shows two examples of miRNA precursor alignments (other sequences were downloaded from miRBase). The comparison of miR-107 precursors among human (*Homo sapiens*), mouse (*Mus musculus*), western clawed frog (*Xenopus tropicalis*), zebrafish (*Danio rerio*), silver carp (*Hypophthalmichthys molitrix*), bighead carp (*Hypophthalmichthys nobilis*), fugu (*Fugu rubripes*) and tetraodon (*Tetraodon nigroviridis*) showed that the 21nt-long miRNA mature sequences are highly conserved in vertebrates, with only one A-U transversion being found at the 3′ end. The same evidence of conservation was noted in the star sequence region, with two mismatches in total. The comparison of let-7 precursors among silver carp (*Hypophthalmichthys molitrix*), zebrafish (*Danio rerio*), human (*Homo sapiens*), fruit fly (*Drosophila melanogaster*) and nematode (*Caenorhabditis elegans*) showed the conservation of let-7 mature sequence between vertebrates (above the horizontal line) and invertebrates (below the horizontal line); while the star sequence, though conserved in vertebrates, was less conserved between vertebrates and invertebrates.

### Highly expressed miRNAs

**Figure 5:**
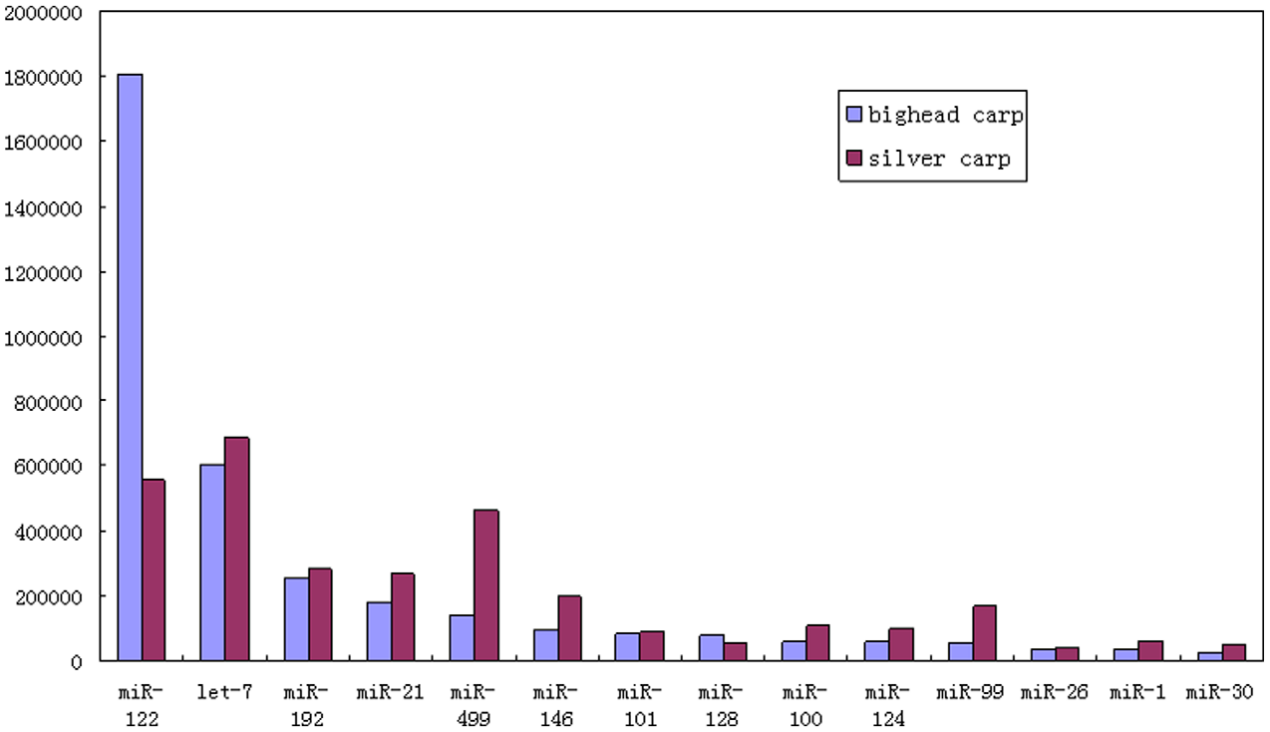
Comparison of the top 14 highly expressed miRNAs in the two carps.

Generally, abundant miRNAs play fundamental and broader regulatory functions. To get a clearer perspective of miRNA expression levels, we have compared 14 miRNA families that have the highest reads numbers in both carps (Figure 5). These 14 miRNA families represent 93% and 87% of the conserved miRNAs in bighead and silver carp, respectively. miR-122 is dominant in bighead carp (1808356 sequences: 48%) and three times lower in silver carp (556153 sequences: 15%). miR-122 belongs to a liver-specific miRNA family which is implicated in fatty acid and cholesterol metabolism and in replication and activation of translation of the hepatitis C virus. This tissue-specific miRNA is also thought to establish patterns of gene expression and may be responsible for maintaining tissues differentiated states [28], [29]. Another highly-expressed miRNA family is let-7, a highly significant miRNA family that was first discovered and characterized in *Caenorhabditis elegans*. This miRNA family plays a role in regulating late developmental events by down regulating *lin-41* [30] and possibly other genes which also contain sequences complementary to the seed region in their 3′ UTRs, possibly as a result of its vital role in developmental timing, let-7 was identified as a highly expressed miRNA in bighead and silver carp (602885 and 687481 sequences, respectively). Ten members of the let-7 miRNA family were characterized by high throughput sequencing, all of which were similar in reads frequency in the two carps (Table S5). miR-499 was one of the muscle-specific and senescence-associated miRNAs. Conversely to miR-122, a greater number of miR-499 reads were detected in silver carp, three times higher than that in bighead carp. Taken together, these 14 miRNAs make up the vast majority of conserved miRNAs in both carps, indicating that they play a significant role in maintaining regular biological processes.

### Analysis of miRNA expression levels in the two carps

A hierarchical cluster analysis of the conserved miRNAs between the two species was performed after their numbers were normalized as TPM (transcripts per million) (Table S6). The result showed that there were five miRNAs with the maximum expression difference in bighead carp and silver carp. The expression level of miR-137, miR-724, miR-7a and miR-734 was more than 10 fold higher in silver than in bighead carp, while for miR-196b, the expression level was more than 10 fold higher in bighead carp than in silver carp.(Table 1).

**Table 1:** miRNAs with the maximum expression difference in bighead carp and silver carp.

miRNA_gene	Bighead carp	Silver carp
miR-7a	1800 (477)	27226 (7552)
miR-137	19 (5)	414 (115)
miR-196	292 (77)	2 (1)
miR-724	138 (37)	2535 (703)
miR-734	4 (1)	130 (42)

Number in parentheses is the normalized data.

**Figure 6:**
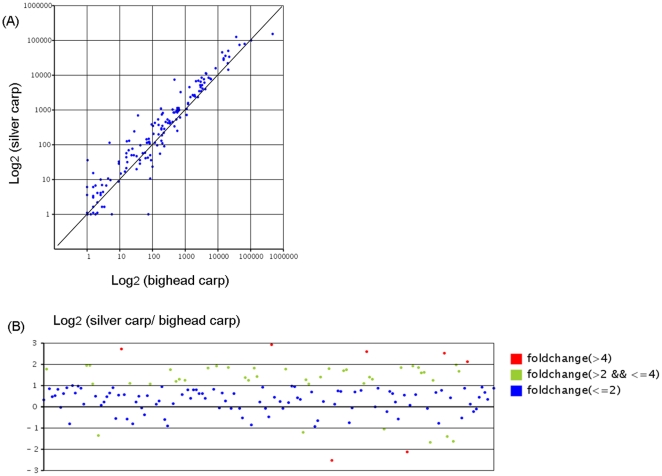
Comparative profiling of the miRNA expression in the two carps. (A) Scatter plot map for miRNA expression levels in bighead carp and silver carp. Each plot represents an individual miRNA. It reflected the proportion of miRNAs that have greater number in bighead carp and miRNAs that have greater number in silver carp, respectively. (B) Log2 ratio of Conserved miRNA in silver carp versus reads in bighead carp. Each plot represents an individual miRNA. Blue plot: equal or less than 2-fold change; green plot: greater than 2-fold change while less than 4-fold change; red plot: greater than 4-fold change.

A scatter plot map comparing the expression patterns of the conserved miRNAs in bighead and silver carp was built using TPM normalized data. In Figure 6A, each dot represents an individual miRNA. Dots above the diagonal indicate the miRNAs whose read number was higher in silver than in bighead carp, while dots below the diagonal indicate less frequent miRNAs in silver than in bighead carp. Figure 6B illustrates differential fold change of miRNAs between the two carps. The fold change was determined by the log2 ratio of reads number of silver carp versus reads number of bighead carp, and the ranges were marked by different colors. Most of the dots were scattered between 1 and -1 (blue dots), representing equal or less than 2-fold changes, which indicated that the majority of miRNAs did not have distinct expression differences between the two carps. Like the blue dots, most of the green dots (between 2-fold change and 4-fold change) and red dots (greater than 4-fold change) were distributed above the zero horizontal line, indicating that most miRNAs with a high expression difference had more sequences in silver carp than in bighead carp.

### Changes in miRNA family members

Three miRNA families, miR-27, miR-30 and miR-181, were analyzed to determine gain and loss of miRNA family members and changes in their sequences (miRNA sequences were downloaded from miRBase). Different members of these miRNA families were presented in different lineages ranging from urochordates to mammals, as we can see in Table 2. miR-27 was not found in sea squirt or any other invertebrates, indicating that the miR-27 gene likely originated in fishes. Up to now, five members of the miR-27 family have been found. Three members have been found in pufferfish (*Fugu rubripes* and *Tetraodon nigroviridis*), four members in bighead carp and silver carp, and five members in zebrafish, indicating that different miRNA family members occur in different species of the same lineage. Two members have been found in clawed frogs and mammals and only one member in chicken, suggesting that gene loss events have happened during evolution of vertebrates. Similar gene loss events were also observed in the miR-30 family in the lineage of teleosts. Five members were found in zebrafish, one member was lost in the two carps and two members were lost in pufferfish. But the missing members were retained in other superior lineages. The situation of gain and loss of family members was observed for miR-181 family as well, suggesting that similar events could be found in other miRNA families.

**Table 2:** miR-27, miR-30 and miR-181 family members in different lineages.

	miR-27 family	miR-30 family	miR-181 family
Human	hsa-miR-27a hsa-miR-27b	hsa-miR-30a hsa-miR-30bhsa-miR-30c hsa-miR-30dhsa-miR-30e	hsa-miR-181a hsa-miR-181bhsa-miR-181c hsa-miR-181d
Bovine	bta-miR-27a bta-miR-27b	bta-miR-30a bta-miR-30bbta-miR-30c bta-miR-30dbta-miR-30e bta-miR-30f	bta-miR-181a bta-miR-181bbta-miR-181c bta-miR-181d
Mouse	mmu-miR-27a mmu-miR-27b	mmu-miR-30a mmu-miR-30bmmu-miR-30c mmu-miR-30dmmu-miR-30e	mmu-miR-181a mmu-miR-181bmmu-miR-181c mmu-miR-181d
Chicken	gga-miR-27b	gga-miR-30a gga-miR-30bgga-miR-30c gga-miR-30dgga-miR-30e	gga-miR-181a gga-miR-181b
Xenopus	xtr-miR-27a xtr-miR-27b	xtr-miR-30a xtr-miR-30bxtr-miR-30c xtr-miR-30dxtr-miR-30e	xtr-miR-181a xtr-miR-181b
Zebrafish	dre-miR-27a dre-miR-27bdre-miR-27c dre-miR-27ddre-miR-27e	dre-miR-30a dre-miR-30bdre-miR-30c dre-miR-30ddre-miR-30e	dre-miR-181a dre-miR-181bdre-miR-181c
Bighead carp	hno-miR-27a hno-miR-27bhno-miR-27d hno-miR-27e	hno-miR-30b hno-miR-30chno-miR-30d hno-miR-30e	hno-miR-181a hno-miR-181bhno-miR-181c
Silver carp	hmo-miR-27a hmo-miR-27bhmo-miR-27d hmo-miR-27e	hmo-miR-30b hmo-miR-30chmo-miR-30d hmo-miR-30e	hmo-miR-181a hmo-miR-181bhmo-miR-181c
Fugu	fru-miR-27b fru-miR-27cfru-miR-27e	fru-miR-30b fru-miR-30cfru-miR-30d	fru-miR-181a fru-miR-181b
tetraodon	tni-miR-27b tni-miR-27ctni-miR-27e	tni-miR-30b tni-miR-30ctni-miR-30d	tni-miR-181a tni-miR-181b
Sea squirt	none	none	cin-miR-181

has: *H. sapien*; bta: *B. Taurus*; mmu: *M. musculus*; gga: *G. gallus*; xtr: *X. tropicalis*; dre: *D. rerio*; hno: *H. nobilis*; hmo: *H. molitrix*; fru: *F. rubripes*; tni: *T. nigroviridis*; cin: *C. intestinalis*.

**Figure 7:**
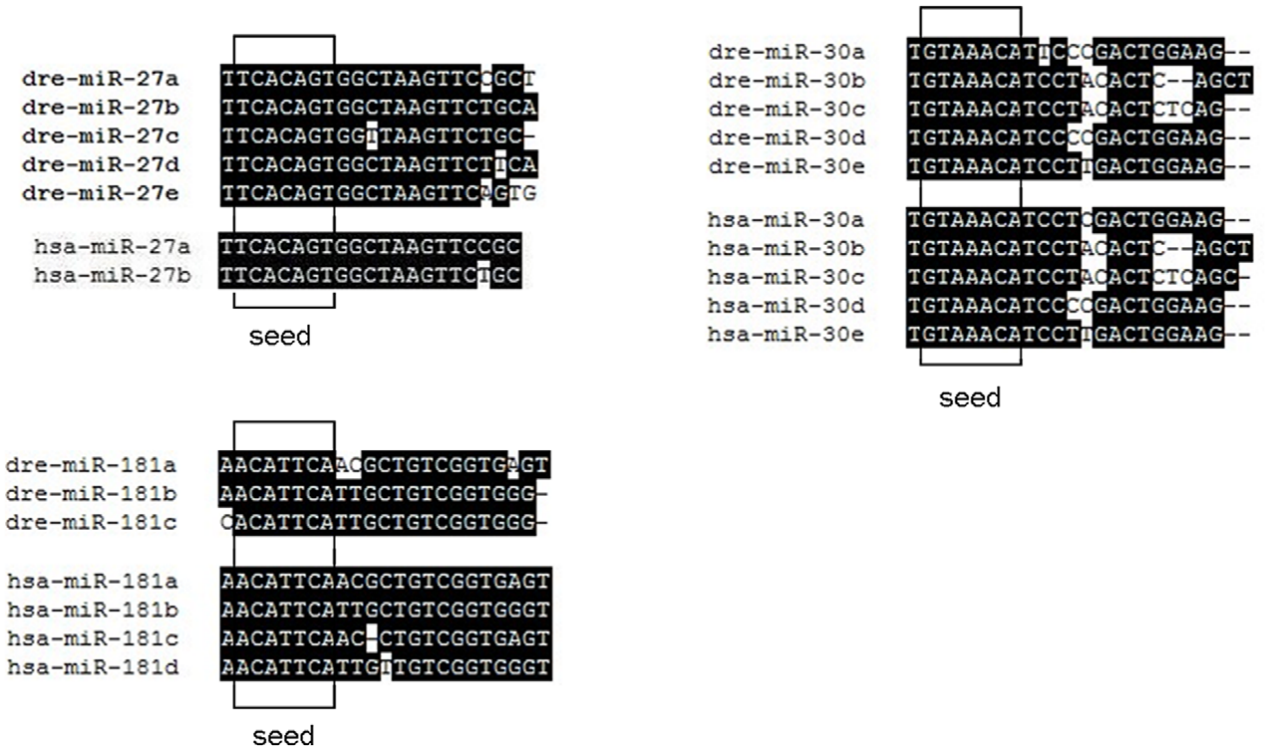
Sequence comparison of miR-27, miR-30 and miR-181 family members in zebrafish and human. Alignments were performed by mega 4.0. dre: *D. rerio*; has: *H. sapien*.

Sequence changes were detected in various miRNA family members, but rarely occurred in the seed region. Alignments were carried out within miR-27, miR-30 and miR-181 family of zebrafish and human, respectively (Figure 7). Although sequence alteration existed in each of the three families, their seed regions were conserved, both in and among the lineages. As a consequence of the minor sequence differences, members within a family had overlapping target sets.

## Discussion

High Throughput Sequencing technologies detect known and novel miRNAs and can also open doors to directly show differences in expression levels. It is widely believed that changes in gene expression patterns underlie many phenotypic differences within, and between, species. In our study, 167 conserved miRNAs were found in bighead carp and 166 in silver carp. Most of these miRNA exist in both carps. The majority of differences were found in the expression level rather than in the sequence conservation of miRNAs. Bighead carp and silver carp exhibit subtle differences in physiology, which is in line with the high number of conserved miRNA expression patterns. A previous study showed that variation in miRNA expression contributes to the differences in physiology, and that the greater the variation in miRNA expression, the larger the differences in physiology [31]. The most striking physiological difference between bighead carp and silver carp is seen in the structure of the head, so we speculate that changes in the miRNA expression (like miRNAs listed in Table 1) might associate with the structure of the head.

In our analysis of miRNA expression in the two carps, some conserved miRNAs showed expression differences, which is in agreement with other studies [31]. Therefore, these studies indicate that the correlation between sequence conservation and expression conservation is weak. But, the majority of conserved miRNAs follow a trend of conserved expression between bighead carp and silver carp.

Gene duplication has long been thought to be a major evolutionary event that allows for emergence of genetic novelty. The fate of duplicated genes is determined by the interaction of three fundamental forces: mutation, genetic drift and natural selection. The most obvious fate is that one of the duplicates is silenced through deleterious mutations and becomes a pseudogene or disappears from the genome entirely. Some gene pairs are “subfunctionalized” and lose complementary functions, so that both genes are maintained in the genome in order to fulfill the complete function of the ancestral gene. The other fate of duplicated genes is that one copy retains its original function while the other becomes “neofunctionalized” acquiring a new adaptive function which is maintained by natural selection [32], [33]. A recent study demonstrated that plant miRNA families are evolving through duplication events similar to those that drive the evolution of protein-coding genes, and that the duplicated copies may acquire divergent expression patterns likely as a result of neo- and subfunctionalization [34]. We speculated that some of the molecular mechanisms might exist in animals as well. Alteration to a duplicated copy of a miRNA gene may impact on its targeting capability, leading to increased or decreased regulatory capacity. Otherwise, one of the miRNA genes might sustain a mutation that changes its targeting capability and drift, while the other would retain its ancestral form and present as conserved animal miRNAs. Under the right circumstances, the mutated duplicate might become favorable selected and eventually fixed in the form of a new miRNA gene. This might be an explanation to that some miRNA family members were lost in one lineage and regained in another lineage (Table 2).

miRNA recognizes its target through the complementarity between seed region and the 3′UTR of target gene. Inspection of miRNA families reveals a predominant trend in which duplicated miRNA genes are most similar in their seed regions (Figure 7). However, it should be noted that any change along the length of the mature miRNA is likely to be of some functional impact. That might be an explanation to that most miRNA duplicates only shift their target spectra modestly via changes to the sequence out of the seed region.

It is very interesting that miR-33 was previously found in mammals (human, rat, mouse, gorilla, etc.), amphibians (xenopus), urochordates (sea squirt) and several invertebrates (fruit fly, mosquito, limpet, etc.), but not in fish. We have discovered two homologs of the human miR-33 in silver carp, their sequences were highly conserved, especially in the seed region (Table S7). This is the first report of miR-33 in fish, and we postulate that the spatial and temporal expression of miRNAs may explain the inability of finding miR-33 in other fish studies; or, it may only exist in silver carp. Both forms are expressed at low level, indicating that fish miR-33 may play a secondary role in gene expression regulation in the fish lineage.

The miRNA transcriptome profiles of the two carps show similar expression patterns and conserved miRNAs account for the majority of expression. It is worth mentioning that most of the newly generated miRNAs identified by the two strategies mentioned above seem to be weakly expressed. Their reads numbers range from tens to hundreds compared to the millions of total reads (see Additional file, table S2 & S3). The same situation was observed in Drosophila [35], suggesting that new miRNA genes are weakly expressed whereas conserved miRNA genes are highly expressed. Wu et al. [36] hypothesized that miRNAs may have dual functions: in tuning and buffering gene expression. In expression tuning, miRNA can modify the mean expression level of their target genes, while in buffering they merely reduce the variance around a preset mean. Wu et al. conclude that new miRNAs are not likely to improve fitness by resetting the mean expression levels of many target genes when they emerge. Instead, they may gain an advantage in homeostasis by reducing gene expression variance. The tuning functions would evolve subsequently and gradually after the new miRNAs are integrated into the genome. So, it seems that they are likely to survive only when the fitness effects are neutral or positive. Subsequent mutations may allow a miRNA to shed target genes that should not be repressed. While the target pool is being shuffled, the expression level of the regulation miRNA may gradually increase.

## Materials and Methods

### Total RNA preparation

For the purpose of obtaining the whole miRNA transcriptomes, we extracted RNAs from five organs (heart, liver, brain, spleen and kidney) in each of the two carps using TRIzol reagent (Invitrogen, Carlsbad, CA, USA). After an examination to assess the quality of RNA by means of electrophoresis and a BioPhotometer plus 6132 (eppendorf, Germany), RNAs of the same species from different organs were mixed together, each with equivalent concentration. Total RNA was extracted according to the manufacturer's protocol.

### High throughput sequencing

Small RNAs, of 16–30 nt in length, were first isolated from the total RNA by size fractionation with 15% TBE urea polyacrylamide gel (TBU) and were ligated to an activated 5′adaptor. After the purification of the small RNA/5′adaptor products, a 3′adaptor (Illumina) was ligated, then purified. The 5′ small RNA adapter is necessary for amplification of the small RNA fragment. This adapter also contains the DNA sequencing primer binding site. The 3′ small RNA adapter is necessary for reverse transcription and corresponds to the surface bound amplification primer on the flow cell used on the Cluster Station. Afterwards, reverse transcription followed by PCR was used to create cDNA constructs based on the small RNA ligated with 5′ and 3′ adapters. The amplified cDNA constructs were purified on 6% Novex TBE PAGE gel and then used for sequencing by the Illumina Genome Analyzer (GPL9330) at the Beijing Genomics Institute, Shenzhen.

### Identification of conserved miRNAs

We first filtered low quality reads, no 3′adaptor reads, 5′adaptor contaminants and sequences shorter than 18 nucleotides. The remained sequences were mapped to zebrafish genome by SOAP (http://soap.genomics.org.cn) with a tolerance of one mismatch. The matched sequences were blasted against Rfam database and NCBI genbank database to separate out rRNA, tRNA, snRNA and snoRNA. After being classified into different categories based on the sequence similarity, the remnant reads of our datasets were compared to currently released miRNAs in miRBase to identify conserved miRNAs [27].

### Two computational strategies to detect novel miRNAs

#### Strategy 1 for detecting novel miRNAs

Using the MIREAP software, a computational tool specially designed to identify potential miRNAs from deep sequencing small RNA libraries, we mapped the unidentified portions of small RNAs to the genome of zebrafish and screened out candidate miRNA sites. A minimal stringent criterion was then used to select miRNA candidates which would ensure that the majority of known miRNAs were recovered, with only a few exceptions whose structures could not satisfy the common features of a miRNA gene. The possible candidate miRNA-miRNA* duplexes must satisfy the following criteria: the putative mature sequence must reside at the stem region and its size was limited to 20–24 nt; the frequency of putative mature sequence should not below 5; The folding free energy of the stem-loop structure was limited below -18 kcal/mol; the maximum tolerance of a bulge size was 4 nucleotides; the maximum size of difference between miRNA and miRNA* was 4 nucleotides; the minimal and maximum size of space between miRNA and miRNA* was 5 nucleotides and 35 nucleotides; the sequence asymmetry between miRNA and miRNA* could not exceed 5 nucleotides.

#### Strategy 2 for detecting novel miRNAs

Based on the biogenesis features of miRNA, we adopted the perl script of stringent criterion written by Wei et al. [37] with slight modifications:

1. The dominant strand must have ten or more reads in the small RNA library not only because miRNAs with a weak expression level would possibly to have no star form in the library, but also because of the authenticity of their existence;
2. The length of the strands of the duplex should both be between 18 and 24 nucleotides long;
3. No more than four mismatches should be allowed, G:U pairing was accepted;
4. The size of a bulge in the candidate should be no more than 4 nucleotides.

In order to satisfy the requirement of input sequences analyzed by mfold, we joined the two sequences in each candidate pair using a standard hairpin-forming linker sequence (GCGGGGACGC). Those pairs that met the following conditions were analyzed further. The folding free energy of the stem-loop structure was limited to below -21 kcal/mol.

### Amplification of known miRNAs precursors

The total DNA of the two carps was extracted from muscle tissues using the phenol/chloroform extraction procedure. Primers were designed by Primer Premier 5.0. Amplification proceeded with a primary denaturation step at 94°C for 5 min, followed by 30 cycles of denaturation at 94°C for 30 sec, annealing at 52°C for 30 sec, and extension at 72°C for 30 sec, with a final extension of 7 min at 72°C. Amplification products were sub-cloned into PMD18-T vector (Takara, Dalian, Liaoning, China) and sequenced.

### Analysis of miRNA expression levels in the two carps

All data were normalized in TPM (transcripts per million). If the number of a miRNA is zero in one of the two carps, it will be changed to 0.01 during the comparing analysis; if the number of a miRNA is less than 1 in both of the two carps after normalization, it will be discarded during comparing analysis.

## Supporting Information

Table S1 Conserved miRNAs in bighead carp and silver carp.
(XLS)

Table S2 Novel miRNAs predicted by strategy 1 in bighead carp and silver carp.
(XLS)

Table S3 Novel miRNAs predicted by strategy 2 in bighead carp and silver carp.
(XLS)

Table S4 Conserved miRNA*s found in our dataset.
(XLS)

Table S5 Comparison of ten let-7 family members in bighead carp and silver carp.
(XLS)

Table S6 Hierarchical cluster analysis of the conserved miRNAs between bighead carp and silver carp.
(XLS)

Table S7 Newly discovered miR-33 in silver carp. Two homologs of the human miR-33 were discovered and sequence alignments were made among several species. hmo: *H. molitrix*; has: *H. sapien*; mmu: *M. musculus*; gga: *G. gallus*; xtr: *X. tropicalis*; cin: *C. intestinalis*; dme: *D. melanogaster*.
(XLS)
